# Does Employee Care Trigger Innovation Under a Healthy and Safe Working Environment? Evidence from the Pharmaceutical Industry in China

**DOI:** 10.3390/healthcare9020194

**Published:** 2021-02-10

**Authors:** Minghui Yang, Qian Lin, Petra Maresova

**Affiliations:** 1 International Business School, Guangzhou College of South China University of Technology, Guangzhou 510800, China; Linqian@gcu.edu.cn; 2 Faculty of Informatics and Management, University of Hradec Kralove, 50002 Hradec Kralove, Czech Republic; petra.maresova@uhk.cz

**Keywords:** employee care, workforce sustainability, occupational health and safety, innovation, pharmaceutical industry

## Abstract

Sustainability of the workforce becomes a crucial issue, of which responsible care for employees can increase job satisfaction and human capital that impact corporate ability to absorb and generate new knowledge. Firms are obligated to provide a healthy and safe working environment for their employees, but it may in turn hinder innovation due to rigid and structured institutional regulations. Drawing on data of 308 China’s pharmaceutical firms from 2010 to 2017, we investigated whether employee care can trigger innovation under corporate adoption of the occupational health and safety management system (OHSMS). Our results suggest that both employee care and OHSMS adoption have a positive impact on innovation. Moreover, the positive relationship between employee care and innovation was more pronounced in firms that had adopted the OHSMS certification. These findings are valuable to policymakers and corporate managers in emerging economies through corroborating the important role of workforce sustainability in facilitating firm innovation.

## 1. Introduction

Innovation is a crucial aspect of any corporate strategy as it is essential to long-term success and to retaining competitive advantage against competitors [1,2]. One of the key components of innovation input is human capital, where staff and workers remain motivated, enabling them to both absorb and produce new knowledge, and then to convert that knowledge into new products and services to improve business processes and technologies. Even if innovation can be triggered by policies such as research tax credits and governmental subsidies in the short run, enhancing the stock of human capital is more effective in the long term [3,4,5]. According to Hall’s study, more than 50% of research and development (R&D) expenses are normally paid to salaries of highly skilled and experienced workforce [6]. Even so, reinforcing human capital is achieved not only by monetary compensation, but also through non-monetary measures such as non-executive incentive schemes [4], legally mandated amelioration [7], employee protection, and employee treatment programs [5,8].

In fact, innovation is a complicate human process driven by technically qualified, skilled, and well-trained people. To support innovation, firms should create workplace environments that are perceived by employees to be pleasant, low-stress, supportive of continuous learning, and encouraging of active sharing of new knowledge and creative ideas [9,10]. Responsible care for employees can increase job satisfaction and help firms to attract and retain talented people who will be the critical factor in corporate innovation process [11,12,13]. In recent years, being socially responsible for employees, and more broadly, the corporate social responsibility (CSR), of which labor care is seen as a key ingredient, have been increasingly emphasized by modern enterprises [14,15].

On the other hand, innovation is also highly uncertain, and cannot be achieved without a corporate culture that is built on long-term strategies and without employees who are committed to their firms. A growing number of firms have adopted the occupational health and safety management system (OHSMS) in order to enhance employee health and mitigate safety risks in the workplace [16]. Some internationally recognized standards such as the Occupational Health and Safety Assessment Series (OHSAS) 18001:2007 and the ISO 45,001:2008 are commonly adopted by firms to avoid workplace accidents [17,18,19], decrease material losses and interruption in manufacturing processes [20], and yield improved financial performance [21]. Nevertheless, extant studies have also argued that management system adoption may in turn hinder innovation due to rigid institutional regulations and a risk-adverse corporate climate [22,23].

There are few “employee care–innovation” relationship studies focused on Chinese companies, despite the fact that firms in China have begun to recognize the importance of responsible employee treatment and their positive outcomes [24,25]. In addition, employee care and occupational health and safety (OHS) are insufficient in the Chinese context [8]. For instance, there were 1.83 million employment dispute cases reported in 2018, together with 2.18 million involved employees, revealing about 0.28% of total employment [26]. With respect to the OHS accidents, there were 23,497 occupational diseases cases and 34,672 death tolls of safety accidents reported in 2018 [27]. In particular, the pharmaceutical industry is ranked in the forefront among all sectors in terms of the numbers of workplace injuries, occupational illnesses, and fatalities [28].

To address the paucity of research in these areas, this study was designed to investigate whether employee care can trigger innovation under the OHSMS, with a sample of pharmaceutical firms in China. First, we examined the association between employee care and firm innovation on the basis of the employee aspect CSR score obtained from the Hexun database, which is the most trusted source of CSR evaluation in China [15]. Second, we evaluated whether corporate adoption of an OHSMS standard (either OHSAS 18,001 or ISO 45,001) had an impact on innovation. Third, we assessed the moderating effect of OHSMS adoption on the relationship between employee care and innovation.

Our study sought to make contributions in several ways. First, while many prior studies have investigated innovation through market factors [29], internal firm characteristics [30], or corporate strategy [31], few studies have shed light on the impact of employees on innovation, specifically from a workforce sustainability perspective. Second, limited research addressed whether OHSMS impacts firm innovation. Third, as pharmaceuticals more often assign intensive resources to innovation such as R&D in medication [15], our study provides empirical evidence to this particular sector, which still largely remains unexplored in previous research.

The rest of the paper is organized as follows. Section 2 presents the literature review and hypotheses, Section 3 demonstrates the methods, Section 4 reports the results and unfolds the discussion, and Section 5 concludes the study.

## 2. Literature Review and Hypotheses

### 2.1. Innovation in Pharmaceutical Industry

**Figure 1:**
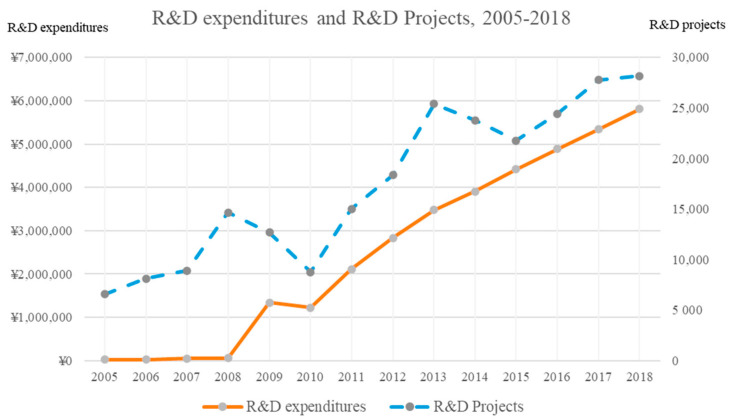
R&D expenditures and R&D projects for pharmaceutical sector in China, 2005–2018. Source: National Bureau of Statistics of China (2019).

In part due to the influence of technologies, different industries have various forms of innovation patterns and technological changes [32,33], and firms in different sectors respond to uncertainties in a variety of industry-specific patterns [34]. The pharmaceutical industry has a long-standing record of innovation over the past 200 years and firms in this sector are always research-intensive. According to the Organization for Economic Co-operation Development (OECD)’s Classification of Manufacturing Industries into Categories based on R&D Intensities [35,36], the pharmaceutical industry is one of the five top high-tech sectors, leading R&D in both direct (e.g., technological development) and indirect (e.g., technological use) applications. In recent years, pharmaceutical firms in emerging economies have invested substantial R&D costs related to medication and clinical tests [37]. Taking China as an example, the R&D expenditures spent across pharmaceutical industries increased from 281.09 million to 58.09 billion Chinese yuan between 2005 and 2018. Similarly, the number of R&D projects carried out in pharmaceutical sector increased from 6581 to 28,167 over the same years [26]. The details of R&D expenditures and projects for pharmaceutical sector in China between 2005 and 2018 are shown in Figure 1.

Although little research is available on the factors related to innovation within the pharmaceutical industry, some studies of internal factors are available including, for example, investigations of R&D expenditure [38,39], technological trajectories [35], life cycle management [40], and access to knowledge sources [41]. There are also some studies on external factors such as the impact of regulation and governance structure, and market environment [42,43,44]. However, in these studies there is little empirical evidence regarding the impact of employees on innovation for pharmaceutical firms. Furthermore, the existing research mainly used survey data to measure corporate innovation, while such cross-sectional survey-type study may result in endogeneity problems that probably generates biased conclusions [45].

### 2.2. Employee Care and Innovation

In the context of CSR research, there has been a focus on the human resource management field since 2017. Scholars claimed that CSR practices will benefit the corporation over time, and thus can be treated as determinants of organizational effectiveness [46]. As a reflection of such effectiveness, corporate innovation is expected to act in a socially responsible manner, enabling the offering of responsible solutions for the momentous challenges of the current era [47]. There are many studies that investigated how innovation is triggered by socially responsible practices, specifically CSR. For instance, a prior study by Abrunhosa and Moura E Sá [48] concluded that socially responsible practices positively influence product innovation. A similar study of service firms demonstrated a positive association between CSR practices and radical service innovation [49]. Sen et al. [50] argued that CSR is a way to strength stakeholder relationship, enabling firms to gain access to valuable resources such as knowledge and funds that pertain in the network of stakeholders [51].

Corporations can effectively develop robust, positive relationships among workers, supervisors, and managers by viewing employee welfare as an important, socially responsible, and necessary practice [52,53]. Providing a comfortable working environment can increase employees’ job satisfaction and encourage them to share new ideas and knowledge [11,13,54]. Survey-based research supports the positive influence of employee on innovation. For example, a survey conducted in 50 large companies in Taiwan concluded that motivational factors such as reciprocal benefits, knowledge self-efficacy, and enjoyment in helping others can improve employees’ knowledge-sharing attitudes and intentions, thus enhancing firms’ knowledge-sharing performance [55]. Another survey study drew on 305 manufacturing and service firms in Germany and revealed that CEOs’, managers’, and non-managerial employees’ ideas and their involvement in innovation process may facilitate innovation performance [56]. Despite the growing academic attention toward the relationship between employee care and innovation [4,5,8], few studies identify whether corporate adopting of OHSMS can strengthen such a relationship.

### 2.3. Management System Adoption: Does it Hinder Innovation?

Management system certifications, such as the ISO 9001, ISO 14,001, OHSAS 18,001, and ISO 45,001, can better administrate inter-related parts of the businesses environment that improve firms’ socio-ecological performance [21,57]. There was a focus regarding whether the adoption of management system can trigger firm innovation in extant research, yet this debate was still open between two opposing opinions [10,23,58]. Supporters suggest that management system can facilitate innovation of both products and processes [45,59]. According to He and Shen [45], firms with management system certification often have better resources management and present more favorable performance in technological innovation. Additional research by López-Mielgo et al. found that the adoption of quality management standards can positively influence firms’ innovation capabilities [60].

Other researchers reached opposite conclusions, finding that management system can negatively affect innovation [61,62,63]. Even if most of management systems follow the concept of continuous improvement, it may hardly trigger innovation because certification is characterized as formal, structured, and liner [22]. It may create a safe climate in which workers are more risk-averse and failure-avoidable, thus contradicting with innovation characterized as adventurous [23]. Song and Su [64] found that certification processes can result in a sense of rigid and bureaucratic regulation, which may in turn reduce companywide creativity. Over the past decade, corporate innovation studies mainly examined management systems related to quality management such as the ISO 9001 and environmental management such as the ISO 14,001 [10,23,45], whereas little research addresses OHSMS such as the OHSAS 18,001 and ISO 45,001.

To conclude literature review, we display a list of extant studies relating to the topic of the current study below in Table 1.

### 2.4. Hypotheses Development

In a direct way, employee care may affect innovation through inter-unit knowledge transfer [9,65]. Moreover, the OHSMS adoption may indirectly influence innovation through effective resource management [45,66]. We also investigated the moderating effect of OHSMS adoption on the relationship between employee care and innovation.

First, innovation is a complex process that largely relies on human capital [1,6]. Employee care may play crucial role in facilitating innovation because employees may actively find new ideas and create knowledge if they have high levels of job satisfaction [11,13,54]. According to the knowledge-based view, firms’ ability of innovation is derived from the control and use of valuable knowledge [51,67]. By providing training and learning opportunities, employees will be more experienced, skilled, and technically qualified, thus leading to better knowledge transfer across different segments within the firm [55,68]. A pleasant working environment also helps companies to attract and retain talented people whose participation and team-working are closely associated with the successful innovation [12]. Novel knowledge can be triggered when employees have greater perceived efficacy and working enthusiasm [69]. Accordingly, the first hypothesis is constructed as follows:

**Hypothesis** **1.** *Employee care has a positive impact on innovation.*

Second, innovation uses resources that in turn requires effective resource management [45]. The resource-based view suggests that rare and valuable resources can produce corporate competitive advantages [70]. Adopting OHSMS helps firms increase health at work, decrease workplace accidents, refine the operational processes, and renovate the manufacturing process, thus leading to better resource utilization [71]. Most of the OHSMS standards follows the plan–do–check–act (PDCA) process and the continuous improvement concept. In keeping with such a trail–feedback–evaluation scenario, technological upgrading and innovative opportunities may be possibly identified [45,72]. The OHSMS adoption may also encourage a long-term oriented corporate culture [57], therefore fostering a never-ending innovation climate under long-term resource allocation strategy. Consequently, the second hypothesis is formulated as follows:

**Hypothesis** **2.** *The adoption of OHSMS has a positive impact on innovation.*

Third, innovation requires sufficient human and financial supports from different stakeholders [51,73]. Providing broad and specific employee benefits and protections can help firms acquire sufficient external resources such as knowledge while the sharing and exchange of such external knowledge may supplement firms’ pool of internal knowledge that facilitates innovation [8,74]. The adoption of OHSMS can also strengthen a firm’s ability to acquire these external resources. The OHSMS standards can be viewed as a tool to deepen corporate stakeholder relationship [17,57]. Standard-adopting companies can charge premium prices for socially concerned customers, thus having more possibility to obtain greater market share and superior financial performance [71]. A good corporate image may be also built, enabling firms to gain access to greater opportunities residing within stakeholder network that supports innovation [6,51]. Taken together, the third hypothesis is proposed as follows:

**Hypothesis** **3.** *The positive impact of employee care on innovation is more significant in firms with the adoption of OHSMS.*

## 3. Research Method

### 3.1. Sample and Data Collection

Our study sample includes pharmaceutical firms listed on either the Shanghai or Shenzhen stock exchanges in China. To access firms’ patent data, we first extracted patent information from the China Stock Market and Accounting Research database (CSMAR), a commonly used financial database that provides China’s capital market information for academia. To verify that no patent data were omitted in the sample, we manually retrieved firms’ registered names through the website of the China’s State Intellectual Property Office (SIPO) for firms with no patent records in the CSMAR. Second, data reflecting corporate care for employee were collected from the Hexun database, which is one of the largest CSR information providers. Third, the data of OHSMS adoption were drawn from the National Certification and Accreditation Information Public Service Platform (CAIPSP), which provides information regarding whether Chinese enterprises have been certified with a particular management system standard. Fourth, the data on other financial performance indicators were obtained from the CSMAR.

The sampling procedure had three stages. First, we identified 346 firms with an exclusive code of “Pharmaceutical Manufacturing” according to the “Industry Classification Guideline” mandated by the China Securities Regulatory Commission (CSRC). Second, we removed firms with no employee-aspect CSR score in the Hexun database, which uses a disclosure-oriented evaluation framework. Simply put, a firm with considerate employee care but without corresponding CSR information disclosure still gains nothing in its employee aspect CSR score. Third, we eliminated firms that had no prior certification records (e.g., ISO 9001 or ISO 14,001) in the CAIPSP because we could not confirm that such firms had been fully retrieved by the CAIPSP due to renaming of the firm or other reasons.

After matching patent data with the previous datasets, we arrived at the final sample of 308 listed pharmaceutical firms. We also dropped observations over the last 2 years to overcome the truncation problem of patent data [75]. According to China’s Patent Law, there is at least an 18-month period for a publicly recognized patent to complete the process from initial filling to final disclosure. On the basis this, we limited the sample to the period of 2010–2017 with 2464 firm-year observations.

### 3.2. Variable Measurements

#### 3.2.1. Measuring Innovation

Patent-based data has been widely used to measure firm-level innovation across different sectors or contexts including the argi-food industry [76], family firms [77], and the green energy industry [78]. For the pharmaceutical sector, patents are a vital strategic weapon for two reasons. First, given the extreme high R&D costs but very low drug-related manufacturing costs, very few pharmaceuticals are willing to input substantial investment in R&D activities without patent protection. Second, patents enable pharmaceutical firms to price their new drug at a high economic margin [79]. Following prior corporate innovation studies that widely use patent count as the proxy [4,5,45], we calculated the natural logarithm of one plus the number of patents granted for a firm in a given year. Compared with survey-based data, secondary innovation data such as quantitative count of patents enables researchers to carry out follow-up studies with large samples, resulting in more objective research findings [80].

#### 3.2.2. Measuring Employee Care

To measure employee care, we used employee aspect CSR score from the Hexun CSR database, which has been frequently deployed in prior research [81]. Found in 1996, Hexun is a top-ranked CSR agency in China that offers comprehensive CSR information for listed firms in China [15]. We only focused on the employee dimension, which is scored by 3 second-class indices and 7 third-class indices under the weighted sum approach. To be specific, 7 third-class indices were scored and normalized for obtaining 3 second-class indices, labelled “responsibility performance”, “employee protection”, and “labor care”. These values were subsequently multiplied by the corresponding weight that result in a final score out of 100. The details of the measurement framework of employee care are shown in Table 2.

#### 3.2.3. Measuring OHSMS Adoption

To assess the level of OHS, we measured whether a firm has OHSMS adoption. A dummy variable was created by assigning the value of 1 if the sample firm had adopted either the OHSAS 18,001 or ISO 45,001 standard, and 0 otherwise.

#### 3.2.4. Measuring Control Variables

We introduced some firm-level variables to control for factors that may influence innovation. First, we included corporate financial performance measures, which link with firm innovation [6]. Following other studies [4,8,51], we controlled for firm size, return on assets (ROA), return on equity (ROE), leverage, and sales growth. Firm size was measured as the natural logarithm of total assets. ROA (net income/total average assets) and ROE (net income/total average equity) were used to capture corporate profitability. Leverage is a debt ratio, calculated by book debt divided by total assets. Sales growth is the growth rate of sales revenue from year *t*−1 to year *t*. Second, we controlled for R&D intensity because firms with greater R&D investment will generate more output of patent [5]. R&D intensity is a ratio of R&D expenditure to total sales. For missing R&D expenditure, we assigned a value of zero. The summary of variable measurement is shown in Table 3.

### 3.3. Model Specification

Since the sample includes both time series and cross-sectional data, we used panel data analysis to empirically test the hypotheses. The likelihood ratio test and Hausman specification test were initially run to examine fixed or random effects and determine the most suitable econometric approach. To test Hypothesis 1, we estimated the regression model as follows:

Innovation_i,t_ = β_0_ + β_1_ CSREmp_i,t-m_ + β_2_ Control variables_i,t_ + ε_i,t_, (1)

where Innovation_i,t_ stands for innovation measured by patent count of firm i in year t. CSREmp denotes employee care of firm i in year t-m, and m = 0, 1, Control. variables_i,t_ refers to *Size*, *ROA*, *ROE*, *Leverage*, *Sales growth*, and *R&D intensity* of firm i in year t. ε_i,t_ is the disturbance term. We also introduced 1-year lagged terms for independent variables to avoid endogeneity problems [17].

To test Hypothesis 2, we estimated the regression model as follows:

Innovation_i,t_ = β_0_ + β_1_ OHSMS_i,t-m_ + β_2_ Control. variables_i,t_ + ε_i,t_, (2)

where OHSMS_i,t_ stands for the adoption of OHSMS of firm i in year t. Other notations remain the same as in Equation (1).

To test Hypothesis 3, we estimated the regression model as follows:

Innovation_i,t_ = β_0_ + β_1_ CSREmp_i,t-m_ + β_2_ CSREmp*OHSMS_i,t-m_ + β3 OHSMS_i,t-m_ + β_4_ Control. variables_i,t_ + ε_i,t_, (3)

where CSREmp*OHSMS_i,t-m_ is the interaction term between CSREmp and OHSMS of firm i in year t. Other notations remain the same as in Equation (1).

## 4. Results and Discussion

### 4.1. Descriptive Statistics

Table 4 reports the descriptive statistics of all variables used for the entire sample. For dependent variable, Innovation shows 1.3333 in the standard deviation, suggesting that there is an apparent difference in the numbers of patents granted across the sample firms. Regarding independent variables, the mean value and median value of CSREmp were 23.2687 and 15.3000, respectively, indicating that the average firm had relatively low degree of employee care. It is striking to note that the standard deviation of CSREmp had the highest value of 24.2848, implying that the difference in employee care was large among sample firms. The mean value of OHSMS was 0.4752, with a median value of 0, revealing that less than half of the observations had adopted either the OHSAS 18,001 or ISO 45,001 standards. With respect to control variables, both ROA and ROE showed negative signs in the minimum value, suggesting that some of sample firms suffered losses over the period analyzed. Sales growth had the highest maximum value of 43.6071, indicating a sharp fluctuation in sales revenue for sample firms. R&D expenditure reported zero in the minimum value, demonstrating that some of sample firms did not spend on R&D expenditure during the analyzed period.

Table 5 shows the correlation matrix for all variables. The results show that Innovation was significantly and positively correlated to CSREmp and OHSMS at the 0.01 significance level. The coefficients fell into the range between 0.1795 and 0.3518, indicating that employee care and OHSMS adoption were both statistically associated with superior innovation performance. In terms of control variables, the correlations between Innovation and Size, ROA, Leverage, and R&D intensity were also significant and positive. However, other control variables including ROE and Sales growth were not found to be significantly related to Innovation.

### 4.2. Empirical Results and Discussions

The fixed effects model is the appropriate econometric approach of the current study in terms of the following criteria. First, the result of Hausman specification test confirmed that the individual effects and the explanatory variables were correlated, and therefore we cannot reject the use of fixed effects model. Second, all independent variables used in this study were time-variant factors, and no other time-invariant factors such as industry and country were involved. We also employed the variance inflation factor (VIF) to examine whether our results were affected by multicollinearity problems. The results showed that the VIF value of all independent variables were far less than the commonly used threshold of 10, meaning that no serious multicollinearity problems were found in our regressions.

To test Hypothesis 1, we examined the impact of employee care on innovation in year *t* and in year *t*−1, respectively. The results shown in Table 6 reveal that over the analyzed period and between firms, *Innovation* enhanced by 1.45 percentage points as a unit increase of CSREmp (*t* = 7.6037, *p* = 0.000). When the one-year lagged term for CSREmp was introduced, we found no statistically significant effect on Innovation (*t* = 0.4972, *p* = 0.6192). The positive coefficient of CSREmp on contemporaneous Innovation indicated that employee care can improve firm innovation, which was consistent with our Hypothesis 1. This finding is in line with previous survey-based research conducted in Taiwan and in Germany [55,56], both of which asserted that good employee treatment contributes to better knowledge-sharing performance and increased level of innovation. Our findings also align with existing empirical evidence from studies of innovation that had a particular focus on employee treatment and non-executive employee stock options [4,5]. However, considerate care for employees may also deter innovation if entrenched managers only use such care for the purpose of private benefits, irrespective of considering corporate goal and the firm as a whole [83].

Our results can be further explained by the knowledge-based view. Caring for employees, such as providing more training and learning opportunities, can promote inter-unit knowledge transfer and new knowledge creation, which are crucial in fostering innovation [11,68]. Firms may also retain and attract talented employees whose knowledge, skills, and experiences are key to success in exploring innovative products. Greater job satisfaction can increase employees’ commitment to firms, frequently leading to greater inventor productivity. They may be inspired to develop creative problem-solving skills in an ongoing culture of innovation. Accordingly, innovation can be facilitated by considerate employee care in a dynamic knowledge-sharing environment.

Table 7 shows results for Hypothesis 2. The positive sign in the coefficient for *OHSMS* indicates that, over the analyzed period and between firms, Innovation increased by 29.06 percentage points among firms with OHSMS adoption (*t* = 2.6628, *p* = 0.008). The results also revealed that the relationship between one-year lagged OHSMS and Innovation was not statistically significant (*t* = 0.8073, *p* = 0.4198), implying that the positive impact of OHSMS standard certification on innovation did not prevail in the long run. These results corroborate that OHSMS adoption leads to better innovation performance, thus supporting Hypothesis 2. Consistent with extant corporate innovation studies that confirmed the positive impact of management system adoption [10,45], our study affirmed the positive effect of the OHSAS 18,001 or ISO 45,001 standard certification on innovation. Nevertheless, our findings are not in line with Castillo-Rojas et al. [23], who showed that the certification of both the ISO 9001 and ISO 14,001 standards may hinder innovation process. Our empirical evidence is also inconsistent with Terziovski and Guerrero [63], wherein the implementation of ISO 9000 standard did not have a signification impact on product innovation.

Some prior research suggested that management system adoption may cause higher level of formalization and stricter control that harm creativity [64]. The creation of a more healthy and safer climate may also lead to organizational bureaucracy and rigidity, negatively affecting new idea creation and innovation ability [23]. However, our findings support the positive influence of OHSMS adoption, which can be explained by the resource-based view. Specifically, certifying with an OHSMS standard can normalize business processes, helping firms better manage valuable resources that can be invested in innovation activities [71]. Furthermore, most of OHSMS standards adhere to the continuous improvement concept, and those standard-adopting firms may have more opportunities to upgrade their existing facilities and identify technological opportunities [45]. Hence, the adoption of OHSMS can facilitate innovation if sufficiently resourced.

With respect to Hypothesis 3, the results shown in Table 8 demonstrate that the estimated coefficients on CSREmp and OHSMS were 0.01585 (*t* = 6.2665, *p* = 0.000) and 0.3827 (*t* = 2.8918, *p* = 0.0000), respectively, which were all significantly positive for Innovation. Yet, the interaction term of CSREmp and OHSMS, namely, CSREmp*OHSMS, was not significantly related to Innovation (*t* = 0.9168, *p* = 0.3596). When the one-year lagged terms for independent variables was introduced, both *CSREmp* and *OHSMS* had significant and positive effects on Innovation, with coefficients of 0.0065 (*t* = 2.1485, *p* = 0.0321) and 0.3355 (*t* = 2.2376, *p* = 0.0256), respectively. The one-year lagged CSREmp*OHSMS was also significantly and positively related to Innovation, with a coefficient in 0.0098 (*t* = 2.5556, *p* = 0.0108), indicating that compared with firms without OHSMS adoption, standard-adopting firms had a 0.98 percentage points increase in *Innovation* as a unit increase of CSREmp. Our finding was consistent with Hypothesis 3, which proposes that the positive impact of employee care on innovation is more pronounced in firms with OHSMS adoption. Even so, it is interesting to notice that the interaction term only positively affected subsequent firm innovation, rather than contemporaneous innovation. This result was similar to that reported by Abad et al. [17], who found that the impact of OHSMS adoption on firm performance indicators is a gradual process, of which the positive influence still exists in the long term. Moreover, when analyzing the lagged independent variables across different models, we found that the impact of employee care on subsequent innovation was not statistically significant for firms without OHSMS adoption, yet such impact was significantly positive for firms with OHSMS adoption.

The significant influence of OHSMS adoption on the association between employee care and innovation can be further explained by firms’ deeper relationship with different stakeholders. Through certifying with an OHSMS standard, firms may gain access to valuable external resources residing within the stakeholder network, thus supporting innovation. Standard adopting firms can provide a positive signal to socially concerned stakeholders, helping them to acquire sufficient knowledge and financial resources that have been characterized as critical factors in maintaining innovation. However, our findings suggest that such reputational and moral capital does not take an immediate effect on innovation. Instead, a good corporate image through OHSMS adoption may gradually influence firm innovation in the long run under better impression management [84]. As proposed by Yang and Maresova [57], OHSMS adoption is a tool to communicate with stakeholders, enriching firms’ internal pool of knowledge by the share and exchange of stakeholders’ knowledge, thus strengthening the positive effect of employee care on subsequent innovation performance through dynamic knowledge transfer. By drawing from the stakeholder relationship perspective, our study complements previous corporate innovation studies that are solely based on the knowledge-based view [51] or resource-based view [8,45].

### 4.3. Robustness Check

Despite the fact that patent count is commonly used in measuring innovation, such simple count number is imperfect in terms of reflecting the quality of innovation, overlooking patents’ technological or economic significance [4]. For robustness, the citation count, computed by the natural logarithm of one plus the number of citations generated by patent filed by a firm in a given year, was used as the alternative dependent variable. As shown in panel A of Table 9, no major variances could be found after using alternative variable, indicating that our results were robust to the use of patent count as proxy for firm-level innovation.

The second robustness check related to the econometric model due to the count nature of the dependent variables. Alternatively, we performed the Poisson regression model in which the number of patents was discrete with non-negative value. Because of the excessive zeros in the number of patents, the zero-inflated Poisson regression may be deployed if the value of the Vuong test was greater than the threshold of 1.96, or the Poisson regression model was used otherwise. As presented in panel B of Table 9, the results remained unchanged after utilizing the alternative statistical approach.

## 5. Conclusions

The main objective of this study was to investigate an inconclusive yet popular debate regarding the influence of employee care on firm innovation. In the context of China’s pharmaceutical industry, we examined whether innovation was impacted by employee care and OHSMS adoption, and how OHSMS adoption might moderate the relationship between employee care and innovation. We used a dataset of 308 listed pharmaceutical firms for the period 2010–2017—the results revealed that both employee care measured by employee-aspect CSR score and corporate adoption of OHSMS proxied by the OHSAS 18,001 or ISO 45,001 standard certification had a positive effect on innovation. The positive impact of employee care on innovation was more pronounced in firms that had adopted OHSMS.

Given that prior studies mainly used survey data to measure innovation and investigate its link with employee care, we added to this literature with our study that included the use of secondary patent data to provide empirical evidence and avoid endogeneity problems. Furthermore, we broadened corporate innovation research that considered how OHSMS adoption affects innovation and investigated whether such adoption can strengthen the impact of employee care on innovation, hence extending the extant understanding of “employee care-innovation” relationship.

Our study offers implications for policymakers and corporate managers. With respect to policymakers, we suggest that it is no longer appropriate for emerging economies such as China to prioritize economic development that does not address critical issues of sustainability such as occupational health and workplace safety. In particular, China’s government announced the transition program of “innovation-driven economy” in the 18th National Congress Party in 2012 [85], meaning that responsible innovation has become a crucial national agenda over the last decade. On the basis of our empirical evidence conducted in China, we emphasized the importance of government incentives both for socially responsible corporate practices toward employees and for creating and enforcing OHSMS adoption. Our findings have particularly strong implications for the Chinese pharmaceutical industry, which is both research-intensive and has high rates of OHS accidents. With respect to corporate managers in China or other emerging economies that have rapid economic advancements with sub-standard CSR performances, our findings demonstrate the importance of viewing expenditures related to CSR and OHSMS adoption as capital investments rather than operational costs due to the benefits coming from innovation.

Our study has some limitations. First, we only offer empirical evidence prior to 2018 without considering up-to-date data due to the truncation problem of patent information. We also only included listed pharmaceutical firms, implying an insufficiency to represent broader sectors of corporate innovation. In fact, small- and medium-sized enterprises have more flexible business systems that are more likely to accept innovative changes. Another limitation is the proxy of patent count we used, which is not sufficient to comprehensively capture innovation. Future study can employ process innovation and technological innovation as the proxy and consider the reverse effect in the “employee care–innovation” relationship. In addition, whether the firm adopts OHSMS can hardly measure actual degree of OHS. Given the increasing concerns with employees’ mental health and their well-being [86], future study can include these factors as the proxies of OHS. We only employed data from the pharmaceutical industry in China, limiting the generalizability of the findings. Future studies can also investigate the “employee care–innovation” relationship in other geographic contexts and different industry sectors.

## Figures and Tables

**Table 1:** Extant research relating to the topic of the current study.

Topic	Author	Sample Origin	Main Findings
Innovation in pharmaceutical industry	DiMasi et al. [38]; DiMasi et al. [39];	USA	The R&D costs related to new drugs and biologics play a key role in triggering medical innovation investment.
	Hering et al. [40]	Global	The lifecycle of a medicinal product and its proper management is important for pharmaceutical companies to ensure innovation.
	Casper and Matraves [42]	UK and Germany	National institutional framework impacts innovation, and UK firms have advantages in generating innovative drugs as compared to German firms.
	Matraves [43]	USA, Japan, Germany, France, Italy, UK	R&D and advertising costs exert substantial influences on the formation of market structure in global pharmaceutical industry.
Employee care and innovation	Chen et al. [5]	Worldwide firms in Kinder, Lydenberg and Domini (KLD) database	Firms with good employee treatment schemes generate more and better patents through enhanced level of job satisfaction and teamworking.
	Tong et al. [8]	China	Employee protection positively influences corporate innovation ability, and this positive relationship is more significant in firms with labor unions and political connections
	Chang et al. [4]	Standard and Poor’s (S&P) 1500 firms	Non-executive employee stock options foster corporate innovation through the risk-taking incentives.
	Holman et al. [54]	UK	Job design influences innovation process including idea generation, idea promotion, and idea implementation through the mediating effect of work-based learning strategies.
	Zhao et al. [25]	The Netherlands	Firms with high proportion of workers on fixed-term contracts have better performance in sales of imitative new product but worse in sales of innovative new products.
The role of management system in innovation	Manzani et al. [10]	Morocco	Within the sociotechnical system of quality management system, the ISO 9001 standard positively impacts incremental product innovation and radical product innovation.
	He and Shen [45]	China	The certification of environmental management system such as the ISO 14,001 facilitates technological innovation through the mediating effects of resource management practices.
	Terziovski and Guerrero [63]	Australia	The certification of the ISO 9000 does not have a statistically significant relationship with product innovation performance, but has a positive and significant impact on innovation performance.
	Castillo-Rojas et al. [23]	Spain	Multiple management system certification such as the combination of the ISO 9001 and ISO 14,001 hinder innovation processes, and such hindrance also impacts firms’ intention to implement certification in the future.
	Rennings et al. [58]	Germany	The certification of environmental management system through the EU Environmental Management and Auditing Scheme has a positive impact on environmental process and product innovation.

**Table 2:** Measurement framework of employee care.

First-Class Measures	Second-Class Measures	Third-Class Measures
Employee care (proportion: 1/1)	Responsibility performance (1/3)	Per capita income of employees (4/15)
		Number of training for employees (1/15)
	Employee protection (1/3)	Number of security check (2/15)
		Number of safety training (3/15)
	Labor care (1/3)	Policy of caring for employees (1/15)
		Number of caring activities (2/15)
		Expenditures paid for caring (2/15)

**Source:** Hexun’s corporate social responsibility (CSR) evaluation framework [82].

**Table 3:** Constructs of the variables.

Variables	Measurement
Innovation	The natural logarithm of one plus the number of patents
CSREmp	A score of employee care extracted from the Hexun CSR database
OHSMS	A dummy variable that 1 for firms that have adopted either the OHSAS 18,001 or ISO 45,001 standards; 0 for firms without standard adoption
Size	Natural logarithm of total assets
ROA	Return on assets, measured as net income/total average assets
ROE	Return on equity, measured as net income/total average equity
Leverage	Book debt divided by total assets
Sales growth	The growth rate of sales revenue from year t − 1 to year *t*
R&D intensity	R&D expenditure divided by total sales; 0 for missing R&D expenditure

**Table 4:** Descriptive statistics.

Variables	*N*	Mean	Median	Min.	Max.	SD
Innovation	2464	2.3696	2.4987	0.0000	6.3172	1.3333
CSREmp	2464	23.2687	15.3000	0.2000	100.0000	24.2848
OHSMS	2464	0.4752	0.0000	0.0000	1.0000	0.4997
Size	2464	21.7061	21.6616	19.0316	25.0187	1.0060
ROA	2464	0.0707	0.0663	−0.2976	0.4939	0.0647
ROE	2464	0.1061	0.0953	−0.9111	6.9179	0.2555
Leverage	2464	0.3278	0.3020	0.0000	1.6081	0.2179
Sales growth	2464	0.3065	0.1365	−0.5822	43.6071	2.1504
R&D intensity	2464	0.0345	0.0309	0.0000	0.5261	0.0336

**Table 5:** Correlation analysis.

Variables	Innovation	CSREmp	OHSMS	Size	ROA	ROE	Leverage	Sales Growth	R&D Intensity
Innovation	1								
CSREmp	0.3518 ***	1							
OHSMS	0.1795 ***	0.0827 **	1						
Size	0.3535 ***	0.2211 ***	0.1727 ***	1					
ROA	0.0775 **	0.1644 ***	0.2472 ***	0.0512	1				
ROE	0.0484	0.1478 ***	0.0556	0.0116	0.4836 ***	1			
Leverage	0.0949 ***	−0.0011	−0.1256 ***	0.1209 ***	−0.3545 ***	−0.0460	1		
Sales growth	−0.556	−0.0127	−0.0036	0.0554	−0.0093	0.0004	0.1078 ***	1	
R&D intensity	0.2242 ***	0.0263	0.1510 ***	0.1105 ***	0.1059 ***	0.0213	−0.2014 ***	−0.0482	1

Notes: ** and *** indicate significance at the 0.05 and 0.01 levels, respectively.

**Table 6:** Regression results for Hypothesis 1: impact of employee care on innovation.

Independent Variables	Dependent Variables: Innovation	
CSREmp	0.01451 (7.6037) ***	
CSREmp_t−1_		0.0013 (0.4972)
**Control Variables**		
Size	0.4904 (6.9855) ***	0.4415 (5.0859) ***
ROA	0.49976 (0.5476)	1.1485 (1.0349)
ROE	−0.0498 (−0.3278)	0.1053 (0.6206)
Leverage	0.5223 (2.2197) **	0.3763 (1.4032)
Sales growth	−0.0548 (−3.4157) ***	−0.0753 (−3.1508) ***
R&D intensity	3.9369 (3.3091) ***	3.0280 (2.2739) **
Intercept	−8.9391 (−5.8792) ***	−7.4851 (−3.9631) ***
F-statistics/chi^2^	8.4296 ***	6.3096 ***
Adjusted R^2^	0.5982	0.5727
Durbin–Watson	1.6076	1.6611
Observations	2464	2464

Notes: ** and *** indicate significance at the 0.05 and 0.01 levels, respectively.

**Table 7:** Regression results for Hypothesis 2: impact of occupational health and safety management system (OHSMS) certification on innovation.

Independent Variables	Dependent Variables: Innovation	
OHSMS	0.2906 (2.6628) ***	
OHSMS_t−1_		0.0962 (0.8073)
**Control Variables**		
Size	0.4142 (5.2162) ***	0.4220 (4.5794) ***
ROA	0.4614 (0.4980)	1.2846(1.1883)
ROE	0.0888 (0.5810)	0.0902 (0.5462)
Leverage	0.5745 (2.4075) **	0.4099 (1.5770)
Sales growth	−0.0482 (−2.9571) ***	−0.0716 (−3.0600) ***
R&D intensity	4.7454 (3.8955) ***	3.8903 (2.9763) ***
Intercept	−7.1386 (−4.1966) ***	−7.1169 (−3.5785) ***
F-statistics/chi^2^	8.0386 ***	6.8782 ***
Adjusted R^2^	0.5868	0.5937
Durbin–Watson	1.5464	1.6454
Observations	2464	2464

Notes: ** and *** indicate significance at the 0.05 and 0.01 levels, respectively.

**Table 8:** Regression results for Hypothesis 3: moderating effect of OHSMS adoption on the relationship between employee care and innovation.

Independent Variables	Dependent Variables: Innovation	
CSREmp	0.01585 (6.2655) ***	
CSREmp*OHSMS	0.0029 (0.9168)	
OHSMS	0.3827 (2.8918) ***	
CSREmp_t−1_		0.0065 (2.1485) **
CSREmp_t−1_*OHSMS_t−1_		0.0098 (2.5556) **
OHSMS_t−1_		0.3355 (2.2376) **
**Control Variables**		
Size	0.3909 (5.0920) ***	0.4029 (4.3703) ***
ROA	0.4698 (0.5265)	1.3161 (1.2224)
ROE	−0.0650 (−0.4362)	0.1055 (0.6409)
Leverage	0.5533 (2.4072) **	0.4143 (1.5917)
Sales growth	−0.0502 (−3.1974) ***	−0.0716 (−3.0701) ***
R&D intensity	4.3255 (3.6828) ***	3.8779 (2.9978) ***
Intercept	−6.9722 (−4.2485) ***	−6.8535 (−3.4536) ***
F-statistics/chi^2^	8.9889 ***	6.8850 ***
Adjusted *R^2^*	0.6178	0.5984
Durbin–Watson	1.5817	1.6700
Observations	2464	2464

Notes: ** and *** indicate significance at the 0.05 and 0.01 levels, respectively.

**Table 9:** Robustness check on alternative variables and models.

Alternative Variables and Models	Model (1)	Model (2)	Model (3)
Panel A: using citation count as the proxy of innovation			
CSREmp	0.2751 (4.3459) ***		
CSREmp_t−1_	0.0083 (0.9054)		
OHSMS		0.1067 (2.9426) ***	
OHSMS_t−1_		−0.0373 (−0.4669)	
CSREmp*OHSMS			0.0006 (0.2354)
CSREmp_t−1_*OHSMS_t−1_			0.0089 (0.4907)
Panel B: using the Poisson regression model as the econometric model			
CSREmp	0.1240 (37.6108) ***		
CSREmp_t−1_	0.0031 (0.2124)		
OHSMS		0.9236 (402.7529) ***	
OHSMS_t−1_		0.7054 (190.8323) ***	
CSREmp*OHSMS			0.0256 (50.3270) ***
CSREmp_t−1_*OHSMS_t−1_			−0.0128 (−5.3904)
Vuong	13.7081	15.1432	5.6807

*** indicate significance at the 0.01 levels.

## Data Availability

The data presented in this study are available on request from the corresponding author. The data are not publicly available due to privacy or ethical restrictions.
